# Effects of Structurally Different HDAC Inhibitors against *Trypanosoma cruzi*, *Leishmania*, and *Schistosoma mansoni*

**DOI:** 10.1021/acsinfecdis.2c00232

**Published:** 2022-06-22

**Authors:** Elisabetta Di Bello, Beatrice Noce, Rossella Fioravanti, Clemens Zwergel, Sergio Valente, Dante Rotili, Giulia Fianco, Daniela Trisciuoglio, Marina M. Mourão, Policarpo Sales, Suzanne Lamotte, Eric Prina, Gerald F. Späth, Cécile Häberli, Jennifer Keiser, Antonello Mai

**Affiliations:** † Department of Drug Chemistry and Technologies, Sapienza University of Rome, P. le A. Moro 5, 00185 Rome, Italy; ‡ Institute of Molecular Biology and Pathology, National Research Council (CNR), Via degli Apuli 4, 00185 Rome, Italy; § Instituto René Rachou, Fundação Oswaldo Cruz, Avenida Augusto de Lima, 1715, 30190-002 Belo Horizonte, Brazil; ∥ Institut Pasteur, Université Paris Cité, INSERM U1201, Unité de Parasitologie Moléculaire et Signalisation, 25−28 Rue du Docteur Roux, 75015 Paris, France; ⊥ Swiss Tropical and Public Health Institute, 4002 Allschwil, Switzerland; # University of Basel, Peterspl. 1, 4001 Basel, Switzerland; ∇ Pasteur Institute, Cenci-Bolognetti Foundation, Sapienza University of Rome, P. le A. Moro 5, 00185 Rome, Italy

**Keywords:** HDAC inhibitors, hydroxamates, benzamides, *T. cruzi*, *Leishmania*, *S. mansoni*

## Abstract

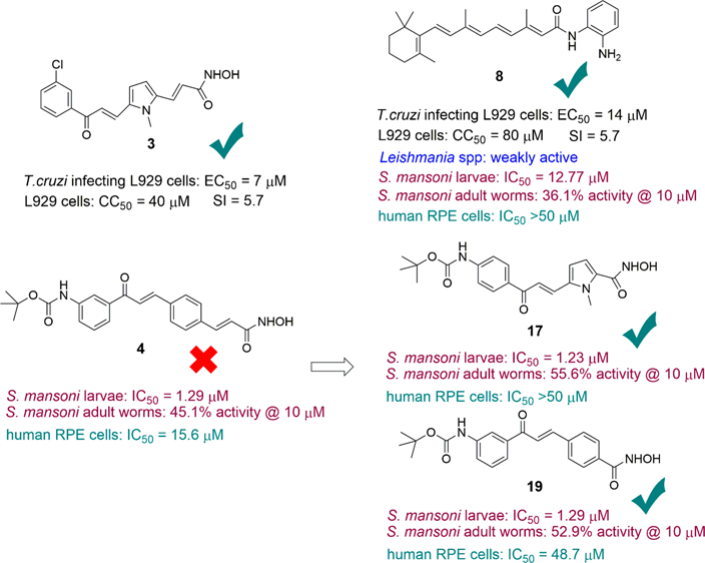

Neglected tropical diseases (NTDs), including trypanosomiasis, leishmaniasis, and schistosomiasis, result in a significant burden in terms of morbidity and mortality worldwide every year. Current antiparasitic drugs suffer from several limitations such as toxicity, no efficacy toward all of the forms of the parasites’ life cycle, and/or induction of resistance. Histone-modifying enzymes play a crucial role in parasite growth and survival; thus, the use of epigenetic drugs has been suggested as a strategy for the treatment of NTDs. We tested structurally different HDACi **1**–**9**, chosen from our in-house library or newly synthesized, against *Trypanosoma cruzi, Leishmania* spp, and *Schistosoma mansoni*. Among them, **4** emerged as the most potent against all of the tested parasites, but it was too toxic against host cells, hampering further studies. The retinoic 2′-aminoanilide **8** was less potent than **4** in all parasitic assays, but as its toxicity is considerably lower, it could be the starting structure for further development. In *T. cruzi*, compound **3** exhibited a single-digit micromolar inhibition of parasite growth combined with moderate toxicity. In *S. mansoni*, **4**’s close analogs **17**–**20** were tested in new transformed schistosomula (NTS) and adult worms displaying high death induction against both parasite forms. Among them, **17** and **19** exhibited very low toxicity in human retinal pigment epithelial (RPE) cells, thus being promising compounds for further optimization.

Neglected tropical diseases (NTDs), such as Chagas disease, leishmaniasis, and schistosomiasis, are a severe problem in underdeveloped regions of the world even today.^1^ Every year, billions of people are suffering from these human parasitic diseases.^2^ Each of them requires new innovative treatments to fight the mortality and morbidity that affect people in large regions of Africa, the Middle East, South America, and Asia.^3^

Chagas disease is caused by the intracellular pathogen *Trypanosoma cruzi*. This etiological agent can be transmitted through vectorial or nonvectorial mechanisms or direct oral transmission.^4^ After a short incubation period, the acute phase of the disease begins and has multiple clinical manifestations like nonspecific viral-like signs, fever, malaise, and lymphadenopathy. Furthermore, patients may manifest irregular transient electrocardiogram problems.^5^ The most effective agents to treat the acute phase of the disease are benznidazole (BZN) and nifurtimox,^4^ but these are highly toxic and have many side effects, and resistance is becoming an issue.^6,7^

Leishmaniasis, which has four different forms of disease (visceral, cutaneous, mucocutaneous, and diffuse forms) is caused by the protozoan parasites of the genus *Leishmania*. The visceral type if untreated is fatal.^8^ Antimonial drugs were used as the first-line treatment of leishmaniasis for decades, but they have several problems, including high toxicity, adverse effects, increasing resistance, high cost, and geographical variability of the response to the treatment.^7,9^ Pentamidine, amphotericin B, and paromomycin are used as second-line treatment drugs but have the same problems.^7^ Also, the potential anticancer drug miltefosine entered the therapeutic arsenal for leishmaniasis; however, it has been reported to have teratogenicity and to induce resistance.^10^

Schistosomiasis, also known as bilharziasis, is caused by Platyhelminths of the genus *Schistosoma*.^11^ The three most relevant species for human infections are *Schistosoma mansoni*, **Schistosoma hematobium**, and *Schistosoma japonicum*.^12^ Schistosome cercariae are able to cross human skin and develop into adult worms, causing severe symptoms, mainly in chronically infected patients, which could result in fibrosis and hepatosplenomegaly.^13^ Currently, there is no vaccine available to prevent human schistosomiasis,^14^ and praziquantel (PZQ) is the only approved drug to treat this infection. PZQ is very effective against adult worms; it is safe and well tolerated with no significant adverse effects.^15^ Unfortunately, it is ineffective on immature, juvenile worms, and drug-resistant forms of the parasite have been described.^16^ Therefore, new drugs able to target multiple stages of the parasite’s life cycle are urgently needed to disrupt the parasite life cycle.

Epigenetic mechanisms and changes in chromatin structure play an important role in parasitic development, and the impact of histone-modifying/interacting proteins is expected to be strong in parasites with complex life cycles and multiple developmental stages. Recent studies have shown the role played by these conserved proteins in the capacity of parasites to adapt to different environments quickly, evade host immune responses, or alter their phenotypes at several critical points of their life cycles.^17−19^ As these features are also common to cancer cells, the use of epi-drugs developed for cancer diseases are considered as a new promising strategy for treating parasitic diseases, also because this piggyback approach can allow faster identification of new lead compounds.^1,20,21^

Histone deacetylases (HDACs) are known to silence critical regulatory pathways, including transcriptional regulation,^22^ cell-cycle progression,^23^ and pro-apoptotic programs.^24^ Among the HDAC inhibitors (HDACi) approved for clinical use in cancer, vorinostat, romidepsin, belinostat, and panobinostat were tested in *Leishmania* at 10 and 20 μM and were ineffective and/or exhibited too high toxicity for macrophages.^25^ Moreover, a benzohydroxamate pan-HDAC inhibitor displayed moderate antileishmanial activity with a narrow selectivity window for human cells,^26^ and the *O*-benzyl derivative of vorinostat was highly potent against the parasites also in in vivo models but could not likely work by inhibiting HDACs. Vorinostat, romidepsin, belinostat, and panobinostat were also tested in *S. mansoni* at 10 μM to determine their effects in schistosomula, adult worm pairs, and egg production with only moderate effects.^25^ On the other hand, several HDACi, either nonselective or SmHDAC8-selective inhibitors, have been reported as active against *Schistosoma*.^27−29^ Nevertheless, the activity observed at the enzymatic level does not always translate into an effect against the parasite, even with SmHDAC8-selective inhibitors. Very recently, a hydroxamate HDACi targeting the unique subpocket of the *T. cruzi*-specific deacetylase *Tc*DAC2 active site has been reported with substantial antiparasitic effects both in cells and in vivo.^30^

Within the A-ParaDDise European project dedicated to the identification of epi-drugs in parasites, we selected a small in-house library of structurally different HDACi by our lab to be tested in phenotypic screenings against *T. cruzi, L. amazonensis*, *L. donovani*, and *S. mansoni*. The compounds were chosen among the uracil-based hydroxamic acids (UBHAs, **1**(31) and **2**), the aroyl-pyrrolyl-hydroxamic acids (APHAs, **3**(32,33)), the *N*-hydroxyphenyl (**4**^34^), *N*-hydroxypyridin-2-yl (**5**^35^), or *N*-hydroxyindol-5-yl (**6**) acrylamides, the oxadiazole-containing hydroxamates (**7**^36^), and the 2′-aminoanilides (**8**^37^ and **9** (entinostat)^38,39^) (Figure 1). They were tested against *T. cruzi* amastigotes and trypomastigotes in immortalized mouse fibroblasts (L929), against *L. amazonensis* and *L. donovani* extracellular promastigotes, *L. donovani* axenic amastigotes, and *L. amazonensis* amastigotes in primary mouse macrophages (high content assay, HCA), and against *S. mansoni* to determine their effects on schistosomula and adult worms.

**Figure 1:**
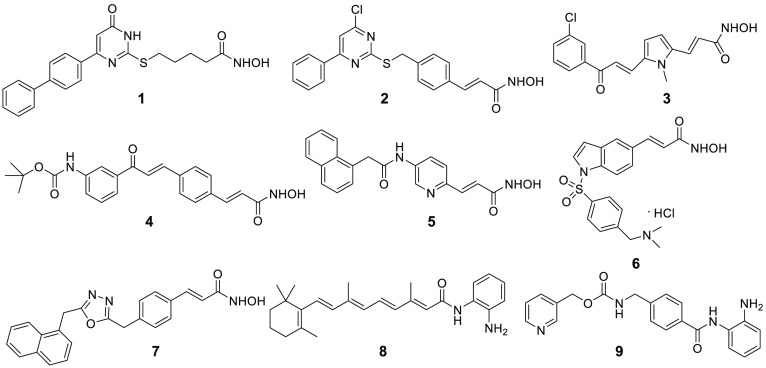
HDAC inhibitors tested against *T. cruzi, Leishmania* spp, and *S. mansoni*.

## Results and Discussion

### Chemistry

**Scheme 1:**
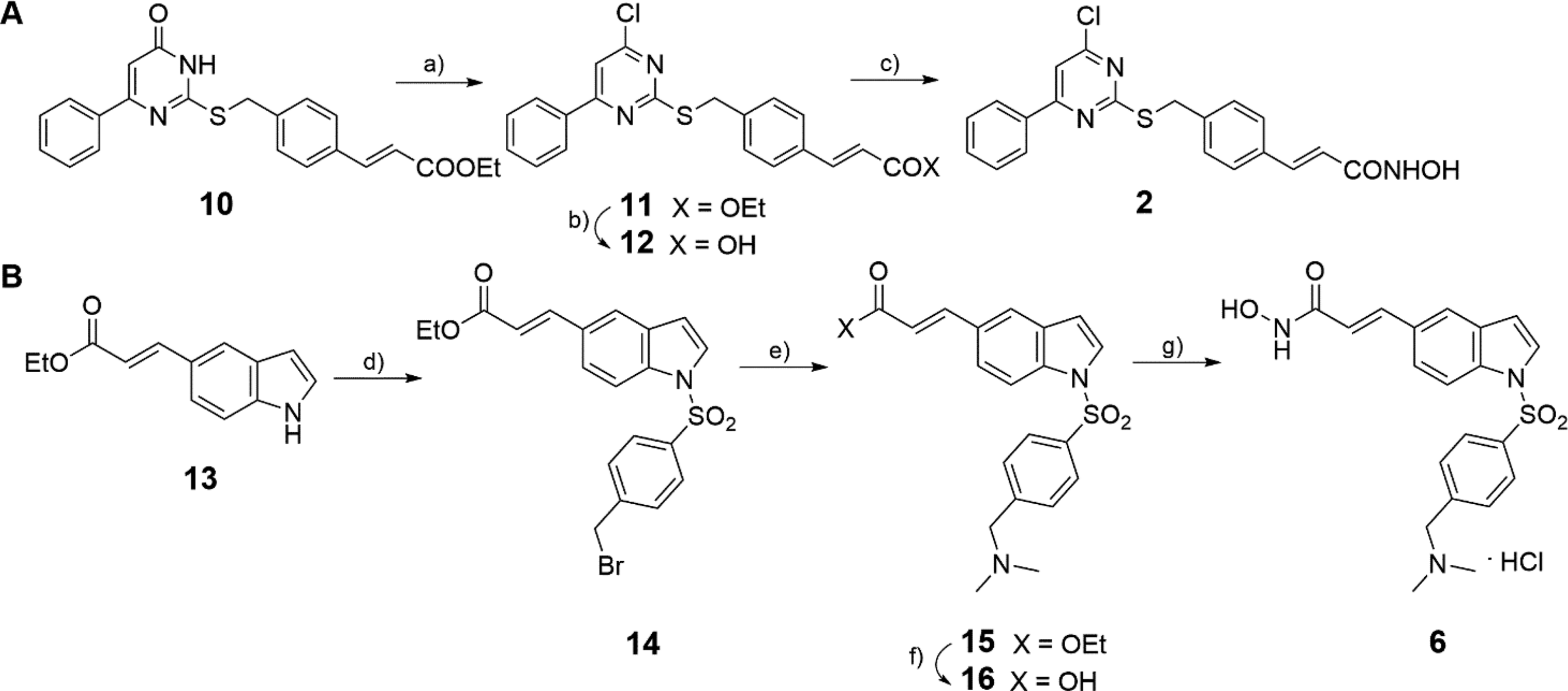
Synthetic Routes for Novel Hydroxamates **2** and **6** Conditions: (a) LiOH hydrate, THF:H_2_O (1:1 v/v), room temperature; (b) 2 N KOH, EtOH, room temperature; (c) (i) ClCOOEt, Et_3_N, dry THF, 0 °C, (ii) NH_2_OC(Me)_2_OMe, room temperature, (iii) Amberlyst 15, MeOH, room temperature; (d) *p*-bromotosyl chloride, NaOH, dry DCE, 0 °C; (e) 2 M *N*,*N*-dimethylamine in THF, EtOH; (f) 6 N HCl, AcOH, 85 °C; (g) (i) Et_3_N, BOP reagent, dry THF, N_2_, (ii) OTHP, dry THF, N_2_, (iii) 2 M HCl in dioxane, dry DCM, N_2_.

The new hydroxamates **2** and **6** were synthesized as outlined in Scheme 1. For the synthesis of **2**, the ethyl 3-(4-(((6-oxo-4-phenyl-1,6-dihydropyrimidin-2-yl)thio)methyl)phenyl)acrylate **10**(40) was treated with phosphorus oxychloride–DMF complex under Vilsmeier–Haack conditions to furnish the 4-chloro ester **11**, which was hydrolyzed with lithium hydroxide into the corresponding carboxylic acid **12**. Compound **12** was further converted into the related hydroxamate **2** by treatment in sequence with (i) ethyl chloroformate and triethylamine in dry THF, (ii) *O*-(2-methoxy-2-propyl)hydroxylamine again in dry THF, and (iii) Amberlyst 15 ion-exchange resin in methanol at room temperature to remove the *O*-protection (Scheme 1A). For the synthesis of **6**, the ethyl 3-(1*H*-indol-5-yl)acrylate **13**(41) was treated with *p*-bromotosyl chloride and NaOH to give the bromomethyl derivative **14**, which underwent nucleophilic substitution of bromine with 2 M *N*,*N*-dimethylamine in THF to furnish the ethyl ester intermediate **15**. The ester **15** was then hydrolyzed with 6 N HCl and glacial acetic acid, and the obtained carboxylic acid **16** was activated with benzotriazol-1-yl-oxy-tris(dimethylamino)-phosphonium hexafluorophosphate (BOP reagent) and triethylamine in dry THF and treated with *O*-(tetrahydro-2*H*-pyran-2-yl) hydroxylamine in N_2_ atmosphere and after with 2 M HCl in dry dioxane/DCM to afford the 1*H*-indol-5-yl hydroxamate **6** (Scheme 1B).

### Biochemical Activities of **2** and **6** against HDAC1–11 Isoforms: Insights on HDAC Inhibiting Activities by the APHA **3**

The new hydroxamates **2** and **6** were screened and profiled via a protease-coupled assay in which the quantification of HDAC enzyme activity is based on the formation of a free fluorescent group, the 7-amino-4-methyl coumarin (AMC).^42^ In particular, **2** and **6** were tested against the 11 human recombinant (hr) HDAC isoforms in 10-dose IC_50_ mode with 3-fold serial dilution starting at 50 μM solutions to determine their inhibitory potency (Table 1). The fluorogenic monoacetylated peptide from p53 residues 379–382 (RHKK(Ac)AMC), in which the AMC moiety required for signal generation is linked to the carboxyl of the acetyllysine that is the target for deacetylation, was used as the general substrate, while the diacetylated peptide from p53 residues 379–382 (RHK(Ac)K(Ac)AMC) was used as substrate for HDAC8, and the fluorogenic class IIa (Boc-Lys(trifluoroacetyl)-AMC) substrate^43^ was employed for class IIa HDACs. After deacetylation by HDACs, the de(trifluoro)acetylated AMC–peptides were sensitive toward lysine peptidase and free fluorogenic AMC was generated and quantified.^42^ SAHA was tested as a reference compound and positive control.

**Table 1:** Inhibition Values (IC_50_, μM) by **2**, **3**, and **6** against the 11 HDAC Isoforms

	IC_50_ (μM)			
HDACs	**2**	**6**	**3**	**SAHA**
HDAC1	1.68	2.07	>300	0.26
HDAC2	4.11	3.49	257	0.92
HDAC3	0.53	0.38	>300	0.35
HDAC4	17.3	16.7	132	0.49
HDAC5	22.9	3.43	131	0.38
HDAC6	0.12	0.018	6.1	0.03
HDAC7	>50	>50	ND^a^	0.34
HDAC8	2.22	1.87	2.0	0.24
HDAC9	>50	31.1	ND	0.32
HDAC10	2.80	2.87	ND	0.46
HDAC11	2.31	4.87	ND	0.36

a ND, not determined.

In 2003, the APHA **3** was reported as the first example of a HDACi 78-fold selective in enzyme assay for the maize HD1-A, a deacetylase homologue of mammalian class IIa HDACs.^32,33,44^ To gain insights into its inhibition profile for human HDACs, we tested **3** against hrHDACs **1–6** and **8** following the above procedure (Table 1). In this case, the 10-dose IC_50_ mode with 3-fold serial dilution starting at 300 μM solutions was used.

The data reported in Table 1 show a general (sub)micromolar inhibition by **2** and **6** for class I, IIb, and IV HDACs and a lower (if any) activity against class IIa HDACs. In detail, **2** and **6** showed a similar behavior toward HDAC inhibition with a preferential, submicromolar/nanomolar inhibition for HDAC3 and HDAC6 and exhibiting a 5–10-fold drop of potency against HDAC4 and -5 and very low or no activity (>50 μM) against HDAC7 and -9. The exception to this rule is the inhibition of HDAC5 by **6**. Indeed, typically the two class IIa HDAC isoforms HDAC4 and HDAC5 show the same level of sensitivity to hydroxamates. Interestingly, **6** was 5-fold more potent against HDAC5 than against HDAC4 (IC_50_^HDAC5^ = 3.43 μM, IC_50_^HDAC4^ = 16.7 μM) and inhibited HDAC5 in the same concentration range of class I HDACs.

The HDAC isoform inhibition profile of **3** was different from those observed with **2** and **6**. In general, **3** was much less effective with a total lack of activity against HDAC1–3 up to 300 μM, very low potency against HDAC4 and -5, and single-digit micromolar inhibition against HDAC6 and -8, which are the real targets of **3** in isolate enzyme assays.

### Effect of **1**–**9** against Amastigote and Trypomastigote forms of *T. cruzi*

Compounds **1**–**9** were tested on mouse L929 fibroblast cells infected with amastigote and trypomastigote forms of *T. cruzi* to detect their capability to inhibit parasite growth (Table 2). Different concentrations of compounds were used until reaching a dose that resulted in a 100% mortality of L929 cells. In parallel, the viability of noninfected L929 cells, expressed as CC_50_ value, was determined, and the selectivity index was calculated when available (Table 2).

**Table 2:** Effect of **1**–**9** on *T. cruzi* Amastigotes and Trypomastigotes Infecting Mouse L929 Fibroblast Cells

		percentage of *T. cruzi* growth inhibition^a^										
lab code	compd	160 μM	80 μM	40 μM	20 μM	10 μM	5 μM	2.5 μM	1.25 μM	EC_50_ (μM)^b^	CC_50_ (μM)^c^	selectivity index (SI)^d^
MC1742	**1**	toxic^e^	toxic	toxic	0	0	0	0	0	inactive	20 ± 0	
MC2129	**2**	toxic	toxic	toxic	3	0	0	0	0	inactive	20 ± 0	
MC1575	**3**	toxic	toxic	90	87	67	39	2	0	7 ± 0.2	40 ± 0	5.7
MC2780	**4**	toxic	toxic	toxic	toxic	toxic	85	0	0	4 ± 0.07	5 ± 0	1.25
MC2590	**5**	toxic	toxic	toxic	toxic	toxic	toxic	toxic	0	inactive	1.25	
MC3031	**6**	toxic	toxic	toxic	toxic	toxic	3	0	0	inactive	5	
MC2059	**7**	toxic	toxic	toxic	toxic	toxic	21	0	0	inactive	5	
MC2392	**8**	toxic	88	76	78	32	7	0	0	14 ± 3.4	80 ± 0	5.7
MS-275	**9**	toxic	toxic	toxic	0	0	0	0	0	inactive	20	
	BZN^f^									3.8	2381	625

a Mean of quadruplicates of a representative assay.

b EC_50_, effective compound concentration that inhibits 50% of the growth of the amastigotes and trypomastigotes. The mean ± SD of at least two independent assays for each compound is reported.

c CC_50_, cytotoxic compound concentration that inhibits 50% of the L929 cell viability. The mean ± SD of at least two independent assays for each compound is reported.

d SI, CC_50_/EC_50_.

e Toxic: L929 death.

f BZN: the reference drug benznidazole.

Among the tested compounds, the UBHA **1**, described as a highly effective antiproliferative agent in human sarcoma stem cells^31^ and later reported as a potent anti-*Toxoplasma gondii* compound,^45^**2**, the 3-(2-pyridinyl)propenyl hydroxamate **5**, selected with **1** as the most potent HDACi in a campaign of epi-drugs against *Plasmodium* parasites,^35^**6**, the 1,3,4-oxadiazole hydroxamate **7**, effective at a low micromolar level against leukemia,^36^ and the 2′-aminoanilide **9** (entinostat), actually in clinical trials for a variety of cancer indications,^46−48^ did not present any effect on the parasite. Compound **4**, known as an effective apoptosis inducer (U937 cells) and antiproliferative agent in cancer cells,^34^ was the most potent against *T. cruzi*, but its high cytotoxicity against noninfected L929 cells (SI: 1.25; Table 2) hampered further development. The APHA **3** and the retinoic 2′-aminoanilide **8**, the latter reported as a context-selective death inducer in acute myeloid leukemia,^37^ displayed micromolar activities against the parasites associated with relatively low toxicity (Table 2); thus, they could be retained as valuable starting points for further studies.

### Antileishmanial Activities of Selected HDACi **1**-**4**, **6**, **8**, and **9** against *Leishmania* promastigotes and amastigotes

Selected HDACi **1**–**4**, **6**, **8**, and **9** were tested at different concentrations against *L. amazonensis* and *L. donovani* extracellular promastigotes and against *L. donovani* axenic amastigotes to determine their effect on free parasites (Table 3). Moreover, they were tested at 10 μM against *L. amazonensis* amastigotes in primary mouse macrophages with the HCA^49^ mimicking the environment in which intracellular amastigotes grow within acidic parasitophorous vacuoles of macrophages (Table 3). This assay coupled the determination of the effect of compounds on parasites within macrophages with an evaluation of their toxicity on host cells.

**Table 3:** Effects of **1**–**4**, **6**, **8**, and **9** against *Leishmania* Promastigotes and Axenic Amastigotes Expressed as a Percent of Inhibition and against Intramacrophagic Amastigotes (HCA)

		percentage of inhibition									
		*L. amazonensis* promastigotes			*L. donovani* promastigotes			*L. donovani* axenic amastigotes			
lab code	compd	20 μM	4 μM	0.8 μM	20 μM	4 μM	0.8 μM	20 μM	4 μM	0.8 μM	HCA
MC1742	**1**	2.1 ± 3.2	NA	NA	NA	NA	NA	29.0 ± 14.0	NA	NA	toxic
MC2129	**2**	83.3 ± 1.8	11.3 ± 1.7	NA	74.8 ± 2.5	2.5 ± 1.4	1.1 ± 2	60.2 ± 1.4	7.8 ± 1.1	16.4 ± 18.6	toxic
MC1575	**3**	9.1 ± 6.3	NA	NA	6.8 ± 1.8	1.7 ± 1.5	3.8 ± 3.4	74.9 ± 1.3	50.8 ± 2.4	4.9 ± 4.9	inactive
MC2780	**4**	97.9 ± 0.4	95.7 ± 0.3	10.5 ± 2.9	95.9 ± 0.3	NA	NA	93.6 ± 0.4	61.4 ± 4.3	39.5 ± 2.0	toxic
MC3031	**6**	60.1 ± 1.7	9.2 ± 3.4	NA	8.6 ± 1.3	6.9 ± 8.7	7.9 ± 7.5	39.6 ± 5.5	0.1 ± 0.8	39.9 ± 2.1	toxic
MC2392	**8**	NA	8.3 ± 3.4	8.1 ± 2.1	6.3 ± 1.7	NA	2.3 ± 1.4	41.8 ± 1.4	5.8 ± 2.5	4.3 ± 1.1	weakly active
MS-275	**9**	NA	NA	NA	NA	NA	NA	28.9 ± 0.9	2.9 ± 10.1	6.7 ± 3.8	toxic

Among the tested HDACi, **1**, **8**, and **9** were practically inactive against free parasites. Compounds **2** and **6** displayed activities only at the highest concentrations, while **3** and **4** inhibited axenic amastigote growth, and **4** also showed inhibition activity down to the micromolar level, thus being the most potent against free parasites. Among the last two compounds, **4** was more effective than **3** in inhibiting parasite growth, but it was expected to be much more toxic (see previous effects on L929 cells) (Table 3). This was confirmed by the HCA assay, in which all tested compounds were toxic for macrophages except **8**, which showed weak anti-*Leishmania* activity at 10 μM and no toxicity toward host cells (Table 3). The moderate potency of **8**, which was only observed on free parasites at 50 μM (data not shown), and the lack of toxicity, likely due to its behavior of context-selective HDACi,^37^ corroborate the relative safety of **8** previously observed in L929 cells.

### Antischistosomal Activities of **1**–**9** against Newly Transformed Schistosomula (NTS) and *S. mansoni* Adult Worms

The HDACi **1**–**9** were screened against newly transformed schistosomula (NTS) at 20 and 10 μM for 72 h to determine their ability to induce death (expressed as percentage of NTS activity). Thereafter, compounds that displayed significant activity (>70% death) against NTS at 10 μM were tested at 1 μM against NTS for 72 h, and the relative compound concentrations that inhibit 50% of the viability of the parasites (IC_50_ values) were determined (Table 4).

**Table 4:** Death Induction in NTS by the HDACi **1**–**9**

		percentage of NTS activity, 72 h			
lab code	compd	20 μM	10 μM	1 μM	IC_50_ (μM)^a^
MC1742	**1**	43.3 ± 1.9	29.3 ± 0.9	ND^b^	>20
MC2129	**2**	67.31 ± 2.9	39.7 ± 0.9	ND	12.89
MC1575	**3**	23.5 ± 0.9	22.3 ± 2.0	ND	>20
MC2780	**4**	100.0 ± 0.0	100.0 ± 0.0	24.0 ± 0.0	1.29
MC2590	**5**	30.8 ± 0.0	31.0 ± 0.0	ND	>20
MC3031	**6**	22.8 ± 1.0	20.9 ± 0.9	ND	>20
MC2059	**7**	39.4 ± 1.0	38.0 ± 1.2	ND	>20
MC2392	**8**	71.1 ± 1.0	37.9 ± 0.0	ND	12.77
MS-275	**9**	42.3 ± 0.0	40.0 ± 2.0	ND	>20

a IC_50_, compound concentration that inhibits 50% of the viability of the parasites.

b ND, not determined.

In this assay, **2** and **8** displayed good activity against the larval forms of *Schistosoma* with an activity of 70% at 20 μM and IC_50_ values of 12.89 and 12.77 μM, respectively. Compounds **1**, **5**, **7**, and **9** exhibited moderate toxicity (30.8–43.3% death induction at 20 μM) (Table 4). Compounds **3** and **6** were less effective, while **4** was the most potent among the tested HDACi, with 100% mortality at 20 and 10 μM and 24% at 1 μM; IC_50_ = 1.29 μM (Table 4).

**Figure 2:**
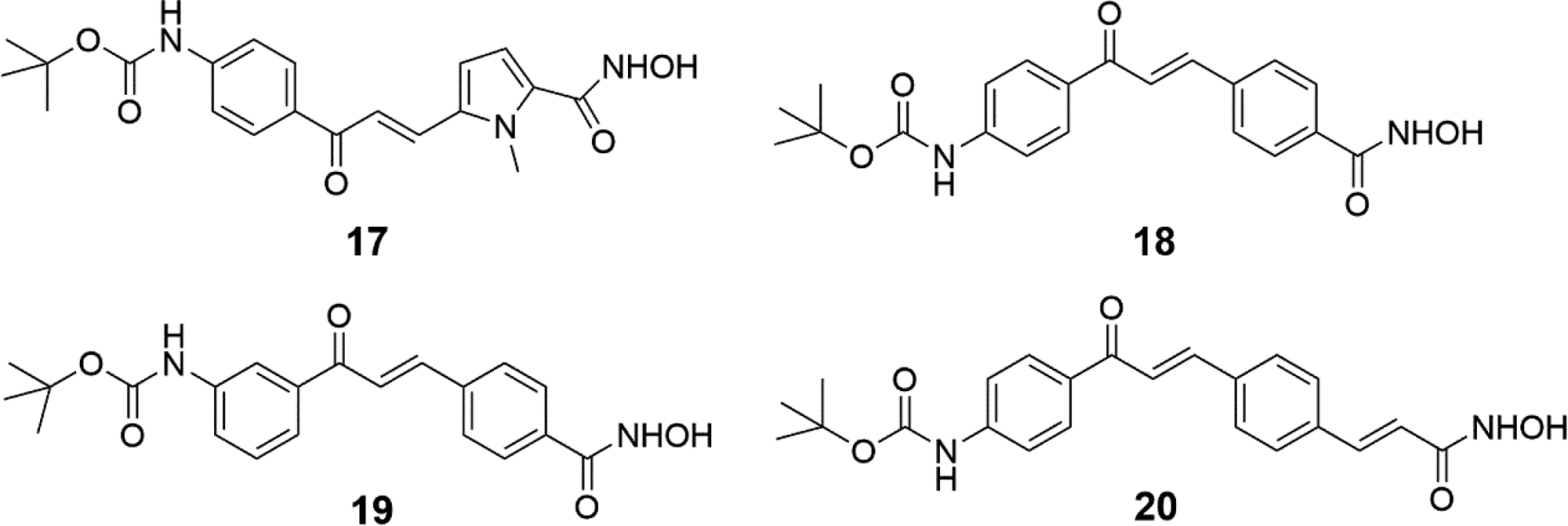
HDACi Analogs of **4** tested in *Schistosoma*.

When tested in cancer cells, some *tert*-butylcarbamate-containing HDACi related to **4** showed lower potencies with regard to antiproliferative and/or pro-apoptotic effects,^34^ suggesting for its analogs a lower degree of toxicity. Due to the considerable potency displayed by **4** against NTS, we selected four strictly related HDACi, compounds **17**–**20** (Figure 2)^34^ to be tested against the parasite (Table 5) with the aim of identifying further active compounds endowed with lower toxicity.

**Table 5:** Effect of **17**–**20** in NTS

		percentage of NTS activity, 72 h			
lab code	compd	20 μM	10 μM	1 μM	IC_50_ (μM)
MC2776	**17**	100.0 ± 0.0	100.0 ± 0.0	28.0 ± 0.0	1.23
MC2779	**18**	100.0 ± 0.0	100.0 ± 0.0	26.0 ± 1.0	1.26
MC2778	**19**	100.0 ± 0.0	100.0 ± 0.0	24.0 ± 0.0	1.29
MC2783	**20**	100.0 ± 0.0	100.0 ± 0.0	22.0 ± 1.0	1.32

By analyzing their chemical structures, **17**–**20** share with **4** the presence of a *tert*-butyl carbamate substituent at the *meta* or *para* position of the common (3-acryloyl)phenyl portion and the hydroxamate function as the HDAC-inhibiting group. The difference is in the central nucleus, which is a pyrrole (**17**), a benzene (**18**, **19**), or a styrene (**20**) moiety. As previously reported,^34^ in cancer cell lines compounds **4** and **17**–**20** behaved differently, with **4** and **20** being the most potent apoptosis inducers (U937 cells) and antiproliferative agents (HCT116, A549, and K562 cells), followed by **17**, less potent in growth arrest assays, and by **18** and **19**, which displayed no or a low apoptotic effect (U937 cells).^34^

In NTS, despite their structural differences and their different potency in cancer cells, all displayed a potent effect of death induction (100% at 20 and 10 μM) with a percentage of activity at 1 μM ranging from 22% to 28% and IC_50_ values in the range 1.26–1.32 μM (Table 5).

Due to their high potency in inducing mortality in NTS, **4** and **17**–**20** were tested in *S. mansoni* adult worms at 20 and 10 μM to determine their activity (Table 6). Also, **8** was tested in adult worms despite its lower efficacy in NTS compared to **4** and **17**–**20** because it was expected to exert low toxicity for human cells (Table 6).

**Table 6:** Percentage of Death Induction by **4**, **8**, and **17–20** in *S. mansoni* Adult Worms

		percentage of activity	
lab code	compd	20 μM	10 μM
MC2780	**4**	63.8 ± 0	45.1 ± 3.9
MC2392	**8**	52.8 ± 2.8	36.1 ± 2.8
MC2776	**17**	58.6 ± 0	55.6 ± 0.0
MC2779	**18**	56.0 ± 7.8	43.1 ± 2.0
MC2778	**19**	58.6 ± 0	52.9 ± 3.9
MC2783	**20**	40.5 ± 2.6	31.3 ± 2.0

Against adult worms, the pyrrole **17** and the *m*-benzoyl hydroxamate **19** exerted the highest effect with over 50% of activity at 10 μM (Table 6).

In parallel, **4**, **8**, and **17**–**20** were tested in the human retinal pigment epithelial (RPE) cell line at 1, 5, 10, 25, and 50 μM using the MTT method to evaluate their effect on cell viability after 48 h of treatment (Table 7). As shown by the IC_50_ values (Table 7), compounds **4**, **18**, and **20** displayed toxicity in RPE cells in the range 15.6–27.2 μM, **19** showed moderate toxicity (IC_50_ = 48.7 μM), while **8** and **17** had a small effect on cell viability only at the highest tested dose. Thus, among the tested HDACi, **8**, **17**, and **19** exhibited a wide selectivity window, and in particular, **17** and **19** were highly potent against *Schistosoma* with very low toxicity in human RPE cells.

**Table 7:** IC_50_ Values of **4**, **8**, and **17–20** in Human Retinal Pigment Epithelial (RPE) Cells

		percentage of RPE cell viability, 48 h					
lab code	compd	1 μM	5 μM	10 μM	25 μM	50 μM	IC_50_ (μM)
MC2780	**4**	75.7 ± 11.4	71.5 ± 12.8	50.2 ± 8.4	40.4 ± 9.8	45.2 ± 6.6	15.6
MC2392	**8**	95.3 ± 28.7	115.1 ± 9.4	90.4 ± 16.7	98.8 ± 4.6	72.0 ± 5.8	>50
MC2776	**17**	74.2 ± 5.2	91.8 ± 11.5	102.5 ± 15.2	91.9 ± 4.5	77.4 ± 2.3	>50
MC2779	**18**	67.9 ± 4.2	73.8 ± 3.3	71.0 ± 7.8	50.3 ± 7.4	43.1 ± 0.1	24.7
MC2778	**19**	75.6 ± 16.9	80.5 ± 13.7	82.0 ± 12.3	68.6 ± 1.3	52.8 ± 16.1	48.7
MC2783	**20**	80.0 ± 11.0	71.6 ± 9.2	63.0 ± 6.0	59.5 ± 2.4	45.6 ± 4.8	27.2

## Conclusions

Most of the antiparasitic drugs for treatment of NTDs are normally quite toxic, and their prolonged use reduces patient compliance and facilitates the emergence of drug-resistant strains. Hence, there is a need for more effective and less toxic alternatives for curing these infections. Histone-modifying enzymes have vital roles in the growth and survival of parasites; thus, targeting the epigenome has been proven to be a good strategy for treating parasitic diseases. Moreover, the large number of alternatives and knowledge built around the epi-drugs would allow the development of cost-effective and reliable alternatives. We tested the structurally different HDACi **1**–**9**, chosen from our in-house library or new compounds, against the kinetoplastids *T. cruzi, Leishmania donovani*, and *L. amazonensis* and the trematode *S. mansoni*. In the *T. cruzi* assay, compound **4** was the most potent in inhibiting amastigotes and trypomastigotes growth (EC_50_ = 4 μM), but it was also highly toxic for L929 cells (CC_50_ = 5 μM); hence, its low selectivity index (SI, 1.25) does not allow further advances. Instead, less potent HDACi such as **3** (EC_50_ = 7 μM) and **8** (EC_50_ = 13.9 μM) displayed lower toxicity (40 and 80 μM, respectively) and are promising starting points for further optimization.

Against *Leishmania* free parasites, **3** and **4** were effective up to low or submicromolar levels on axenic amastigotes, **2** and **6** inhibited parasite growth only at the highest tested dose (20 μM), and **1**, **8**, and **9** were inactive. However, the HCA revealed toxicity on macrophages for most of the tested compounds, no antileishmanial activity for **3**, and weak activity for **8**. Hence, despite its moderate potency, **8** can be considered worthy of further studies in *Leishmania*.

When tested in *S. mansoni* NTS, **4** emerged as the most potent death inducer up to 1 μM. Hence, further HDACi analogs **17**–**20** strictly related to **4** were evaluated and found to have similar potency against both NTS and adult worms with IC_50_ values around 1.2–1.3 μM in larvae and 31–56% activity against adult worms at 10 μM.

Toxicity toward host cells seems to be the limiting factor for the development of HDACi in parasites, most likely due to the high conservation among the catalytic domains of histone-modifying enzymes of most of the organisms. Among our tested HDACi, the compounds with a chance of further development are the HDAC6/8-selective inhibitor **3** (in *T. cruzi*), totally inactive against the nuclear HDAC1–3, the retinoic 2′-aminoanilide **8** (in all tested parasites), which is highly selective for the PML-RARα repressive complex and specifically inhibits the HDACs involved in this cell context, and **17** and **19**, which exerted high potency against NTS (IC_50_ values 1.23 and 1.29 μM, respectively), >50% activity against *S. mansoni* adult worms at 10 μM, and very low toxicity (IC_50_ values > 50 and 48.7 μM, respectively) in human RPE cells.

## Methods

### Chemistry

Melting points were determined on a Buchi 530 melting point apparatus and are uncorrected. ^1^H and ^13^C NMR spectra were recorded at 400 MHz on a Bruker AC 400 spectrometer; chemical shifts are reported in δ (ppm) units relative to the internal reference tetramethylsilane (Me_4_Si). EIMS spectra were recorded with a Fisons Trio 1000 spectrometer; only molecular ions (M^+^) and base peaks are given. All compounds were routinely checked by TLC, ^1^H NMR, and ^13^C NMR. TLC was performed on aluminum-backed silica gel plates (Merck DC, Alufolien Kieselgel 60 F254) with spots visualized by an UV light. All solvents were reagent grade and, when necessary, purified and dried by standard methods. The concentration of solutions after reactions and extractions involved using a rotary evaporator operating at a reduced pressure of ca. 20 Torr. Organic solutions were dried over anhydrous sodium sulfate. Elemental analysis has been used to determine the purity of the described compounds, which is >95%. Analytical results are within ±0.40% of the theoretical values. All chemicals were purchased from Aldrich Chimica, Milan (Italy), or Alfa Aesar, Karlsruhe (Germany), and were of the highest purity.

#### Synthesis of Ethyl 3-(4-(((4-Chloro-6-phenylpyrimidin-2-yl)thio)methyl)phenyl)acrylate (**11**)

A mixture of anhydrous *N,N*-dimethylformamide (0.65 mL, 8.47 mmol) and phosphorus oxychloride (0.79 mL, 8.47 mmol) was stirred at room temperature for 1 h; then, a solution of **10**(40) (1.90 g, 4.84 mmol) in anhydrous chloroform (23 mL) was added. The resulting mixture was stirred at room temperature for 2 h and then quenched with saturated aqueous sodium hydrogen carbonate (80 mL), and the phases were separated. The aqueous layer was extracted twice with fresh chloroform (4 × 80 mL), and the organic extracts were collected, washed with brine, dried, and evaporated to give a residue, which was purified by silica gel column chromatography eluting with a mixture of ethyl acetate/hexane (1:10 v/v) to provide pure **11**. Mp 101–103 °C; yield 75%, recrystallization solvent toluene. ^1^H NMR (CDCl_3_) δ 1.33 (t, 3H, CH_2_C*H*_3_), 4.25 (q, 2H, OC*H*_2_CH_3_), 4.46 (s, 2H, SC*H*_2_), 6.40 (d, 1H, CH=C*H*COOEt), 7.37 (s, 1H, C_5_-*H*), 7.44–7.52 (m, 7H, benzene rings), 7.65 (d, 1H, C*H*=CHCOOEt), 7.98 (d, 2H, benzene ring). MS (ESI), *m*/*z*: 411 [M + H]^+^.

#### Synthesis of 3-(4-(((4-Chloro-6-phenylpyrimidin-2-yl)thio)methyl)phenyl)acrylic Acid (**12**)

A mixture of **11** (500 mg, 1.22 mmol), LiOH·H_2_O (112 mg, 2.68 mmol), THF (4.3 mL), and H_2_O (4.3 mL) was stirred at room temperature for 18 h. The solution was evaporated under reduced pressure, and the residue was poured into water (50 mL) and extracted with ethyl acetate (2 × 20 mL). HCl (2 N) was added to the aqueous layer until the pH was 2, and the resulting solid in suspension was filtered and purified by silica gel column chromatography eluting with a mixture of ethyl acetate/hexane (1:3 v/v) to obtain the desired product **12** by TLC as a pure white solid. Mp 200–202 °C; yield 85%; recrystallization solvent acetonitrile. ^1^H NMR (CDCl_3_) δ 4.47 (s, 2H, SC*H*_2_), 6.40 (d, 1H, CH=C*H*COOH), 7.38 (s, 1H, C_5_-*H*), 7.45–7.52 (m, 7H, benzene rings), 7.72 (d, 1H, C*H*=CHCOOH), 7.99 (d, 2H, benzene ring). MS (ESI), *m*/*z*: 381 [M – H]^−^.

#### Preparation of 3-(4-(((4-Chloro-6-phenylpyrimidin-2-yl)thio)methyl)phenyl)-*N*-hydroxy Acrylamide (**2**)

Triethylamine (2.3 mmol, 233 mg, 0.32 mL) and ethyl chloroformate (2.1 mmol, 226.5 mg, 0.2 mL) were added in sequence to a 0 °C cooled solution of **12** (0.87 mmol, 320 mg) in dry tetrahydrofuran (8 mL), and the resulting mixture was stirred at 0 °C for 10 min. The white salt was filtered off, and *O*-(2-methoxy-2-propyl)hydroxylamine (5.2 mmol, 548 mg, 0.39 mL) was added to the filtrate. The resulting mixture was stirred at room temperature for 1 h and evaporated under reduced pressure, and the residue was diluted in MeOH (3.0 mL). Amberlyst 15 ion-exchange resin (175 mg) was added to the solution of the *O*-protected hydroxamate, and the mixture was stirred at room temperature for 1 h. Afterward, the reaction mixture was filtered, and the filtrate was concentrated in vacuum to give the crude **2**, which was purified by recrystallization from methanol. Mp 180–182 °C; yield 62%; recrystallization solvent methanol. ^1^H NMR (DMSO*-d*_6_) δ 4.53 (s, 2H, SC*H*_2_), 6.42 (d, 1H, CH=C*H*CONHOH), 7.41 (d, 1H, C*H*=CHCONHOH), 7.50–7.60 (m, 7H, benzene rings), 8.00 (s, 1H, C_5_-*H*), 8.21 (d, 2H, benzene ring), 9.05 (s, 1H, CON*H*OH), 10.73 (s, 1H, CONHO*H*). ^13^C NMR (DMSO-*d*_6_) δ 34.5, 116.0, 118.9, 127.4 (2C), 127.6 (2C), 128.5 (2C), 128.9, 129.3 (2C), 133.8, 135.7, 136.3, 141.5, 161.2, 161.8, 165.3, 172.5. Anal. (C_20_H_16_ClN_3_O_2_S) Calcd: C, 60.38; H, 4.05; Cl, 8.91; N, 10.56; S, 8.06. Found: C, 60.56 H, 4.09; Cl, 8.99; N, 10.45; S, 7.97. MS (ESI), *m*/*z*: 398 [M + H]^+^.

#### Synthesis of Ethyl 3-(1-((4-(Bromomethyl)phenyl)sulfonyl)-1*H*-indol-5-yl)acrylate (**14**)

Ethyl 3-(1*H*-indol-5-yl) acrylate **13**(41) (1.71 mmol, 300 mg) was added to a suspension of NaOH (6.84 mmol, 273.60 mg) in 1.2 mL of 1,2-dichloroethane. The mixture was cooled to 0 °C and stirred for 10 min. Then, a solution of *p*-bromotosyl chloride (2.05 mmol, 553.0 mg) in 4 mL of 1,2-dichloroethane was added dropwise over 10 min. After 30 min from the addition, it was left stirring at room temperature for one night. At the end the reaction was quenched with H_2_O (50 mL) and extracted with 1,2-dichloroethane (3 × 50 mL). The combined organic phases were washed with a NaCl saturated solution (100 mL), dried, and evaporated under reduced pressure. The residue was purified in a silica column using AcOEt:*n*-hexane 1:4 to obtain compound **14**. Mp 148–150 °C; yield 81%; recrystallization solvent benzene. ^1^H NMR (CDCl_3_) δ 1.36 (t, 3H, COCH_2_C*H*_3_, *J* = 8 Hz), 4.26–4.31 (q, 2H, COC*H*_2_CH_3_), 4.41 (s, 2H, C*H*_2_Br), 6.44 (d, 1H, C*H*=CH, *J* = 16 Hz), 6.71 (d, 1H, CH=C*H*, *J* = 16 Hz), 7.46–7.48 (m, 2H, aromatic protons), 7.52–7.55 (dd, 1H, aromatic proton), 7.59 (d, 1H, aromatic proton, *J* = 3.6 Hz), 7.70 (d, 1H, aromatic proton, *J* = 3.6 Hz), 7.76 (d, 1H, aromatic proton, *J* = 16 Hz), 7.86–7.89 (m, 2H, aromatic protons), 8.0 (d, 1H, aromatic proton, *J* = 8.4 Hz). MS (ESI), *m*/*z*: 448 [M + H]^+^.

#### Synthesis of Ethyl 3-(1-((4-((Dimethylamino)methyl)phenyl)sulfonyl)-1*H*-indol-5-yl)acrylate (**15**)

A 2 M *N,N*-dimethylamine solution in THF (7.20 mmol 3.60 mL) was added to a solution of ethyl 3-(1-((4-(bromomethyl)phenyl)sulfonyl)-1*H*-indol-5-yl)acrylate **14** (1.59 mmol, 650.0 mg) dissolved in 10 mL of EtOH. The solution was stirred at room temperature until completeness of the reaction and concentrated in vacuo. The residue was purified in a SiO_2_ column using CHCl_3_:CH_3_OH 10:1 as the eluant to obtain compound **15**. Mp 152–154 °C; yield 89%; recrystallization solvent acetonitrile. ^1^H NMR (CDCl_3_) δ 1.35 (t, 3H, COCH_2_C*H*_3_, *J* = 8 Hz), 2.27 (s, 6H, CH_2_N(C*H*_3_)_2_), 3.41 (s, 2H, C*H*_2_N(CH_3_)_2_), 4.26–4.31 (q, 2H, COC*H*_2_CH_3_), 6.44 (d, 1H, C*H*=CH, *J* = 16 Hz), 6.70 (d, 1H, CH=C*H*, *J* = 16 Hz), 7.43 (d, 2H, aromatic protons, *J* = 8 Hz), 7.51–7.54 (dd, 1H, aromatic proton), 7.61 (d, 1H, aromatic, *J* = 4.0 Hz), 7.70 (d, 1H, aromatic proton, *J* = 3.6 Hz), 7.76 (d, 1H, aromatic proton, *J* = 16 Hz), 7.84–7.87 (m, 2H, aromatic protons), 8.01 (d, 1H, aromatic proton, *J* = 8.0 Hz). MS (ESI), *m*/*z*: 412 [M + H]^+^.

#### Synthesis of 3-(1-((4-((Dimethylamino)methyl)phenyl)sulfonyl)-1*H*-indol-5-yl)acrylic Acid (**16**)

A 6 N HCl solution (5.81 mL) was added to a solution of ethyl 3-(1-((4-((dimethylamino)methyl)phenyl)sulfonyl)-1*H*-indol-5-yl) acrylate **15** (1.29 mmol, 480.0 mg) and glacial acetic acid (5.81 mL). The resulting mixture was heated to 85 °C and stirred until completion of the reaction. The solution was evaporated under reduced pressure, and the residue was washed with diethyl ether to obtain after filtration compound **16** as a white solid. Mp 145–147 °C; yield 83%; recrystallization solvent acetonitrile. ^1^H NMR (DMSO*-d*_6_) δ 2.64 (s, 6H, CH_2_N(C*H*_3_)_2_), 4.27 (s, 2H, C*H*_2_N(CH_3_)_2_), 6.53 (d, 1H, C*H*=CH, *J* = 16 Hz), 6.89 (d, 1H, CH=C*H*, *J* = 16 Hz), 7.64–7.72 (m, 4H, aromatic protons), 7.88–7.99 (m, 3H, aromatic proton), 8.12 (d, 2H, aromatic protons, *J* = 4.0 Hz), 10.57 (bs, 1H, CH_2_N(CH_3_)_2_*HCl), 12.37 (bs, 1H, COO*H*). MS (ESI), *m*/*z*: 384 [M – H]^−^.

#### Synthesis of 3-(1-((4-((Dimethylamino)methyl)phenyl)sulfonyl)-1*H*-indol-5-yl)-*N*-hydroxy Acrylamide (**6**)

Triethylamine (0.75 mmol, 0.055 mL) and BOP reagent (0.21 mmol, 93 mg) were added under a nitrogen atmosphere to a solution of 3-(1-((4-((dimethylamino)methyl)phenyl) sulfonyl)-1*H*-indol-5-yl) acrylic acid **16** (0.15 mmol, 58 mg) in anhydrous THF (1 mL). The resulting mixture was stirred for 30 min at room temperature. Subsequently, under a nitrogen atmosphere, *O*-(tetrahydro-2*H*-piran-2-yl) hydroxylamine (0.195 mmol, 22.84 mg) was added. At the end of the reaction, THF was evaporated in vacuo, water was added, and the compound was extracted with ethyl acetate (3 × 50 mL). The organic phase was dried with anhydrous sodium sulfate and evaporated, and the obtained solid was purified on a silica column using CHCl_3_/CH_3_OH (10:1) as the eluant. The solid was added to anhydrous dichloromethane (2.1 mL), and the *O*-protected hydroxamate was cleaved with 2 M HCl in dioxane to obtain the final compound **6**. Mp 195–197 °C; yield 91%; recrystallization solvent acetonitrile. ^1^H NMR (DMSO-*d*_6_) δ 2.63 (s, 6H, CH_2_N(C*H*_3_)_2_), 4.30 (s, 2H, C*H*_2_N(CH_3_)_2_), 6.46 (d, 2H, C*H*=CH, *J* = 12 Hz), 6.90 (d, 1H, CH=C*H*, *J* = 12 Hz), 7.49–7.53 (m, 2H, aromatic protons), 7.80–7.82 (m, 4H, aromatic protons), 7.99 (d, 1H, aromatic proton), 8.11 (d, 2H, aromatic protons), 10.84 (bs, 1H, CON*H*OH), 10.86 (s, 1H, CONHO*H*). ^13^C NMR (DMSO-*d*_6_) δ 44.9 (2C), 63.9, 108.7, 114.7, 119.5, 121.1, 125.5, 127.1, 127.8 (2C), 128.6, 130.2 (2C), 133.0, 134.8, 136.1, 141.9, 143.9, 165.0. Anal. (C_20_H_22_ClN_3_O_4_S) Calcd: C, 55.11; H, 5.09; Cl, 8.13; N, 9.64; S, 7.35. Found: C, 55.37; H, 5.19; Cl, 8.22; N, 9.42; S, 7.11. MS (ESI), *m*/*z*: 436 [M + H]^+^.

### HDAC1–11 Isoforms Inhibition Assay

Individual IC_50_ values for each HDAC isozyme were measured with the homogeneous fluorescence release HDAC assay.^42^ Purified recombinant enzymes were incubated with 3-fold serial-diluted inhibitors starting at 50 μM solutions in 10-dose IC_50_ mode. The deacetylase activities of HDACs 1, 2, 3, 6, 10, and 11 were measured by assaying the enzyme activity using the fluorogenic monoacetylated peptide from p53 residues 379–382 (RHKK(Ac)AMC), the activity of HDAC8 was measured using the diacetylated peptide from p53 residues 379–382 (RHK(Ac)K(Ac)AMC), and the activities of HDACs 4, 5, 7, and 9 (class IIa HDACs) were measured using the fluorogenic class IIa (Boc-Lys(trifluoroacetyl)-AMC) substrate.^43^ Deacetylated AMC–peptides were sensitive toward lysine peptidase, and free fluorogenic 4-methylcoumarin-7-amine (AMC) was generated, which can be excited at 355 nm and observed at 460 nm.^42^ The data was analyzed on a plate-to-plate basis in relation to the control and imported into analytical software (GraphPad Prism 8.0 using the least-squares best-fit method resulting in sigmoidal dose–response curves with variable slope).

### Determination of Cytotoxicity on L929 and RPE Cells

To assess the cytotoxicity over L929 cells, 4000 L929 cells suspended in 200 μL of RPMI-1640 medium plus 10% FBS and 2 mM glutamine were added to each well of a 96-well microtiter plate and incubated for 3 days at 37 °C in a humid 5% CO_2_ environment. The medium was then replaced, and the cells were exposed to compounds at different concentrations. After 96 h of incubation with the compounds, alamarBlue was added and incubated for 4–6 h, and the absorbance at 570 and 600 nm was assessed. Controls including untreated and 1% DMSO-treated cells were run in parallel. Four technical replicates were run on the same plate, and the experiments were repeated at least in two biological replicates. The results were expressed as the percent difference in the reduction between treated (TC) and untreated cells (UT).^50,51^ Linear interpolation was used to determine the CC_50_ values. To assess the cytotoxicity over RPE cells, 15 000 cells suspended in 200 μL of DMEM-F12 medium plus 10% FBS and 2 mM glutamine were added to each well of a 96-well microtiter plate. After 24 h, different compounds were added to the medium at concentrations ranging from 1 to 50 μM for 48 h. Each dose was tested in sextuplicate. MTT was added to each well at a final concentration of 0.5 mg/mL, and after 4 h of incubation at 37 °C, the formazan salt was dissolved with 200 μL of isopropylic alcohol. The absorbance of each well was measured with an ELISA reader (DASIT) at 570 nm wavelength, and the viability was calculated for each concentration of compound used as (OD of treated cells/OD of control cells) × 100. The concentration of compounds that causes 50% of cell viability inhibition (IC_5_0) was also calculated.

### *T. cruzi* Infection Assays in L929 Cells

As previously described,^50,51^ the in vitro test of trypanocidal activity was performed using the *T. cruzi* Tulahuen strain expressing the *Escherichia coli* β-galactosidase gene. Specifically, 4000 L929 cells were added in 80 μL of supplemented medium, without phenol red, to each well of a 96-well microtiter plate. After overnight incubation, 40 000 trypomastigotes suspended in a 20 μL volume were added to the cells and incubated for 2 h. The medium containing parasites that did not penetrate the cells was replaced with 200 μL of fresh medium and incubated for an additional 48 h, allowing the establishment of infection. The medium was then replaced by compounds diluted at different concentrations in fresh medium (200 μL), and the plate was incubated for 96 h at 37 °C. After this period, 50 μL of 500 μM chlorophenol red β-d-galactopyranoside in 0.5% Nonidet P40 was added to each well, followed by an incubation of 18 h at 37 °C, after which the absorbance at 570 nm was measured. Controls included uninfected cells, untreated infected cells, infected cells treated with 3.8 μM BZN (positive control), or cells exposed to 1% DMSO. The results were expressed as the percentage of *T. cruzi* growth inhibition in compound-tested cells as compared to the infected cells and untreated cells. Quadruplicates were run on the same plate, and the experiments were repeated at least in two biological replicates. The compounds and the reference drug BZN were serially diluted (1:2 ratio) in the RPMI medium. Linear interpolation was used to determine the EC_50_ values.

### Antileishmanial Assays with Host-Cell Free Parasites

The antileishmanial activity of the compounds was evaluated using an adapted resazurin reduction assay^52^ with cell-cycling promastigotes from the logarithmic growth phase of *L. amazonensis* and *L. donovani* and with axenic *L. donovani* amastigotes. Compounds were tested in quadruplicate at 20, 4, and 0.8 μM at 26 °C for promastigotes and 37 °C for axenic amastigotes in 384-well plates. DMSO vehicle and AmB (1 μM) were used as controls. Two days later, resazurin was added (10 μL per well at 25 μg/mL), and the fluorescence intensity was measured 24 h later using a Tecan Safire 2 reader (excitation 558 ± 4.5 nm; emission 585 ± 10 nm). Following background subtraction (complete parasite culture medium with resazurin in the absence of parasites), data were expressed as percentages of growth inhibition compared to DMSO-treated controls.

### HCA on Intramacrophagic *L. amazonensis* Amastigotes

The high content assay (HCA) was used to assess the antileishmanial activity against intramacrophagic amastigotes of *L. amazonensis*.^49^ Briefly, after 3 days of coincubation with the compounds, BMDM nuclei and parasitophorous vacuoles (PVs) were stained with Hoechst 33342 (Invitrogen Molecular Probes, 12 μM) and LysoTracker Green (Life Technologies, DND-26, 1 μM), respectively. Acquisition of the images was performed on live cell cultures using the OPERA QEHS using the green (488 nm), blue (405 nm), and red (561 nm) channels for detection of PV, nuclei, and amastigotes. Merged green, blue, and red fluorescence images were acquired with a 10× air objective (NA 0.4). Analysis was performed according to sequential segmentation steps as previously described.^49^

### Newly Transformed Schistosomula (NTS) and Adult *S. mansoni* Worms

The *S. mansoni* lifecycle is maintained at the Swiss Tropical and Public Health Institute (Swiss TPH) as described.^53^ An in-house method was used to obtain cercariae from infected *Biomphalaria glabrata* snails. Briefly, infected snails were isolated in 24-well plates (filled with pond water) and placed under a strong light source for 3–4 h to initiate shedding of the cercariae. Cercariae were collected and mechanically transformed to newly transformed schistosomula (NTS), which were then kept in the incubator (37 °C and 5% CO_2_) in medium 199, supplemented with 5% FCS and 1% penicillin/streptomycin, for at least 12 h to a maximum of 24 h until usage. Adult *S. mansoni* worms were collected by dissecting the mesenteric veins of infected mice at day 49 postinfection. Worms were incubated in supplemented RPMI medium (5% FCS, 100 U/mL penicillin, and 100 μg/mL streptomycin) at 37 °C and 5% CO_2_ until usage.

### In Vitro Phenotypic Screening Assays

For NTS and adult *S. mansoni* worms, transparent flat-bottom 96- and 24-well plates were used, respectively (Sarstedt, Switzerland). The drugs were initially tested at 20 and 10 μM in triplicate on NTS and repeated once; each well contained 30–40 NTS. Phenotypic reference points such as motility, morphology, and granularity were used to score incubated parasites’ overall viability (scores from 0 to 3).^53^ Parasites were judged via microscopic readout 72 h after incubation; compounds that showed high activity (75–100% reduction of viability) against NTS were subsequently tested for additional concentrations for IC_50_ determination (Calcusyn software version 2.0). Identified hits from the NTS screening (>75% activity at 10–20 μM) were tested on *S. mansoni* adult worms. At least three worms (both sexes) were incubated with RPMI 1640 supplemented with 5% (v/v) FCS and 1% (v/v) penicillin/streptomycin at 37 °C and 5% CO_2_ for 72 h at concentrations of 20 and 10 μM.^53^ The experiment was conducted in duplicate and was not repeated; standard deviations were calculated from two wells. For all in vitro assays, negative controls (using the highest concentration of DMSO) were included.
